# Clinical Efficacy and Patient Adherence Hurdles of Remote Patient Monitoring (RPM) Frameworks in Managing Hypertension and Type 2 Diabetes: A Scoping Review

**DOI:** 10.7759/cureus.113642

**Published:** 2026-07-30

**Authors:** Susith C Athukorala

**Affiliations:** 1 Health Informatics, Postgraduate Institute of Medicine, University of Colombo, Sri Lanka, Colombo, LKA

**Keywords:** digital health technologies (dht), hypertension and therapy, interoperability, patient adherence, remote patient monitoring devices, therapeutic interventions, types 2 diabetes

## Abstract

Remote patient monitoring (RPM) frameworks represent a critical paradigm shift in chronic disease management, enabling continuous physiological tracking outside traditional clinical environments; however, large-scale clinical deployment is frequently bottlenecked by operational attrition, patient compliance barriers, and therapeutic provider inertia. This scoping review maps the global evidence regarding the technical configurations, data integration architectures, and human factor friction points that dictate the implementation efficacy of RPM platforms for hypertension and type 2 diabetes. Adhering to the Preferred Reporting Items for Systematic Reviews and Meta-Analyses Extension for Scoping Reviews (PRISMA-ScR) consensus standards, a systematic search was executed across PubMed, Embase, and Scopus, utilizing a hybrid data extraction approach combining manual triage with a local, regular expression-driven text-mining pipeline to chart a final corpus of 400 full-text peer-reviewed source publications, yielding 701 distinct technical and operational evaluation entries. The comprehensive data charting pipeline mapped exactly 701 unique architectural and behavioral evaluation layers across the included literature. Quantitative synthesis indicated that automated upper-arm oscillometric blood pressure cuffs constituted the primary remote monitoring hardware (32.8%; n=230), while continuous glucose monitors/glucometers and wearable sensors demonstrated symmetrical distributions (11.1%; n=78 each). Ingestion architectures heavily favored automated, passive cloud-native uplinks (43.2%; n=303) over short-range active Bluetooth middleware pairing (19.4%; n=136). Implementation barrier cross-tabulation revealed that clinical workflow friction and daily routine disruption constituted the single most prevalent deployment bottleneck (27.1%; n=190). Patient-centric human factors included digital health literacy gaps (15%; n=105) and technology anxiety or alert fatigue (9.3%; n=65), while systemic provider therapeutic inertia was documented in 8.4% (n=59) of settings. Bivariate co-occurrence analysis established that operational workflow friction frequently compounded alongside digital literacy deficiencies (n=10) and clinical inertia (n=7). Ultimately, RPM deployment longevity is directly governed by technical architecture selections and the optimization of downstream clinician workflows. To minimize patient attrition and avoid technical debt, future health informatics implementations must move past isolated device deployments, prioritizing semantic interoperability via Health Level Seven (HL7) Fast Healthcare Interoperability Resources (FHIR) standards.

## Introduction and background

Remote patient monitoring (RPM) technologies, the use of connected digital devices to monitor patient health data from home, have transitioned from novel digital health interventions to essential pillars of modern chronic disease management [1-3]. By leveraging connected medical peripherals, RPM frameworks enable continuous, real-time data collection outside traditional clinical environments, offering an unprecedented opportunity to manage conditions such as hypertension and diabetes mellitus proactively [1,2]. The clinical value proposition of these systems is well-documented; systematically tracking physiological metrics allows for the early detection of clinical deterioration, optimization of pharmacological regimens, and reduction of emergency department utilization [2,4].

However, despite robust clinical evidence supporting the efficacy of remote monitoring, large-scale implementation across healthcare delivery organizations remains fragmented and frequently plagued by high attrition rates [5,6]. While early digital health literature focused predominantly on technological feasibility and accuracy, contemporary deployment failures are increasingly attributed to operational and human factors [5,6]. The introduction of an RPM pathway introduces structural shifts in routine clinical data workflows, creating significant friction points for both patients and healthcare providers [6].

On the provider side, clinical teams routinely experience alert fatigue, increased administrative workloads, and persistent therapeutic inertia (delays in escalating treatment despite recorded clinical need) when attempting to titrate medications based on remotely transmitted data [7,8]. Concurrently, patients frequently struggle with digital health literacy gaps, technology-induced anxiety, and the logistical burden of integrating rigid measurement protocols into their daily routines [9,10]. Furthermore, the lack of seamless architectural interoperability between hardware modalities and proprietary cloud infrastructure often forces reliance on fragmented data synchronization models, compounding operational inefficiencies [4,11].

To systematically address these deployment bottlenecks, it is necessary to chart the intersection between hardware architectures, data synchronization pipelines, and the operational barriers documented in global literature. While narrow systematic reviews have investigated isolated elements of remote care, such as standalone mobile applications or continuous glucose monitoring in specific cohorts, there remains a critical knowledge gap regarding how technical modalities directly influence clinical workflow friction and human factor barriers at scale [1,12].

To address this gap, the primary objective of this scoping review is to systematically map the structural, technical, and behavioral landscape of RPM systems for hypertension and diabetes mellitus. Specifically, this review aims to (1) evaluate the distribution of physiological hardware modalities and data synchronization architectures, (2) identify recurring workflow friction points, digital literacy barriers, and clinical inertia, and (3) establish a data-driven framework to guide health informaticians and clinical leaders in deploying scalable, low-friction digital health interventions.

## Review

Materials and methods

Study Design and Framework

This study was conducted as a systematic scoping review to map the technical and human factor landscape of RPM systems applied to hypertension and diabetes mellitus. The study protocol was structured in accordance with the Preferred Reporting Items for Systematic Reviews and Meta-Analyses extension for Scoping Reviews (PRISMA-ScR) guidelines [1].

Eligibility Criteria and Population, Concept, and Context (PCC) Framework

To ensure a systematic, comprehensive, and transparent mapping of the literature, study eligibility was anchored using the PCC framework: population (P): adult patients (aged ≥18 years) diagnosed with chronic cardiovascular or metabolic conditions, specifically hypertension, type 1 diabetes mellitus, or type 2 diabetes mellitus; concept (C): digital RPM infrastructure, focusing on the interface between physiological hardware modalities, data synchronization pipelines, clinical outcomes, and implementation barriers; and context (C): global healthcare delivery environments, including primary care systems, ambulatory networks, community health initiatives, and home-based digital health interventions.

Search Strategy and Information Sources

Comprehensive literature searches were systematically deployed across primary biomedical and technical databases, including PubMed, Embase, and Scopus through July 2026. The search strategy utilized combinations of Medical Subject Headings (MeSH) terms paired with Boolean logic operators to maximize retrieval sensitivity. Two reviewers independently screened titles and abstracts against predefined eligibility criteria using the PCC framework, followed by independent full-text evaluation. Any screening discrepancies were resolved through iterative joint consensus discussions. The review adheres to the PRISMA-ScR reporting standards.

The systematic approach to data collection involved structured electronic queries across multiple medical and technical literature databases. The explicit database strings, field-specific keywords, and Boolean logic parameters utilized across the primary indexing servers are detailed in Table 1.

**Table 1:** Systematic search strategy, database-specific query strings, and inclusion filtering criteria

Database	Search string/keywords	Filter criteria and limits
PubMed	("Remote Patient Monitoring"[Mesh] OR "RPM" OR "telemonitoring" OR "home telemetry") AND ("Diabetes Mellitus, Type 2"[Mesh] OR "Hypertension"[Mesh] OR "chronic disease management") AND ("Implementation Science" OR "workflow friction" OR "barriers" OR "patient adherence")	English language, peer-reviewed, publication date limits applied
Embase	('remote patient monitoring'/exp OR 'telemonitoring' OR 'home telemetry') AND ('non insulin dependent diabetes mellitus'/exp OR 'essential hypertension'/exp) AND ('clinical workflow' OR 'patient compliance' OR 'barrier')	Article, conference paper, English language
Scopus	TITLE-ABS-KEY(("Remote Patient Monitoring" OR "RPM" OR "telemonitoring") AND ("Type 2 Diabetes" OR "Hypertension") AND ("implementation barrier" OR "workflow friction" OR "adherence"))	Peer-reviewed journals only, English language

The electronic database search was limited to peer-reviewed articles published in the English language.

Study Selection

Articles were included if they evaluated the deployment, structural architecture, data synchronization models, or human factor barriers of digital remote monitoring interventions matching the PCC criteria. Studies evaluating purely inpatient monitoring networks, non-adult cohorts, or non-digital monitoring pathways (e.g., telephone-only voice tracking without peripheral device integration) were excluded.

To ensure a focused evaluation of relevant digital health architectures, strict eligibility parameters were established for literature screening. The complete breakdown of the screening framework is presented in Table 2.

**Table 2:** Documented inclusion and exclusion criteria for study eligibility selection

Selection parameter	Inclusion criteria	Exclusion criteria
Study type	Peer-reviewed scoping reviews, systematic reviews, and original observational studies	Editorials, commentaries, conference abstracts without full text, and white papers
Target conditions	Essential hypertension and type 2 diabetes mellitus	Gestational diabetes, acute hypertensive crises, or secondary forms of hypertension
Technology focus	Remote patient monitoring platforms involving home physiological data transmission	Standalone educational mobile apps without remote clinician data ingestion or monitoring pathways
Outcome metrics	Must explicitly report clinical efficacy, patient adherence barriers, or operational deployment workflows	Studies focusing exclusively on hardware manufacturing costs or bench testing without human clinical trials
Language and scope	Full-text publications available in the English language	Non-English publications lacking verified, peer-reviewed translated text

The structural screening flow balanced strictly across progressive triage phases. A total of 722 records were initially identified through database searches. Following the removal of 14 duplicate records, 708 unique articles underwent independent title and abstract screening. During this primary triage phase, 240 records were excluded due to a lack of alignment with the inclusion criteria. Of the remaining 468 reports sought for full-text retrieval, 68 reports could not be sourced or retrieved. A final pool of 400 articles met all eligibility criteria and were successfully retrieved for full-text evaluation and systematic data charting.

Data Charting and Coded Extraction Matrix

A standardized data charting matrix was developed using an automated Python-driven regular expression (Regex) classification framework to systematically extract contextual fragments from the final pool of 400 articles. Regex patterns were algorithmically engineered in Python using domain-specific keyword dictionaries, word boundary matching, and case-insensitive string variations, followed by iterative sensitivity calibration on a 10% pilot sample to ensure extraction accuracy. The charting pipeline yielded 701 distinct textual records mapping across four targeted thematic pillars: (1) hardware modality, coded for specific remote monitoring device types, including oscillometric upper-arm blood pressure cuffs, standard glucometers, continuous glucose monitors (CGMs), and consumer or medical-grade wearable sensors (e.g., smartwatches, fitness trackers); (2) data synchronization model, tracked the architectural method of data ingestion, including active Bluetooth-to-application middleware hub pairing, direct device-integrated cellular networks, passive automated cloud/server uplinks, or manual web portal and computer-interface inputs; (3) clinical endpoints, documented primary physiologic targets, specifically blood pressure indicators (systolic and diastolic blood pressure, absolute variations in "mm Hg") and glycemic metrics ("HbA1c" percentage values, fasting plasma glucose); and (4) workflow and implementation barriers, characterized systemic implementation friction points, specifically workflow/routine disruption, digital health literacy gaps, technology-induced anxiety or alert fatigue, and therapeutic or clinical inertia.

To support descriptive quantitative synthesis and meet the charting transparency expectations of PRISMA-ScR Item 12, raw text fragments were programmatically coded into binary variables (1=metric present; 0=metric absent). Textual formatting anomalies and column alignment shifts caused by unquoted internal punctuation within article metadata were audited and programmatically repaired to ensure absolute cross-tabulation accuracy.

Granular data validation patterns, binary target variables, and algorithmic coding details are compiled within the supplementary data spreadsheet cataloged in the Appendices.

Results

Selection and Characteristics of the Included Studies

The systematic screening workflow successfully captured the target literature distribution. The initial electronic database search yielded 722 records, which were reduced to 708 unique records following duplicate exclusion (n=14). Title and abstract triage eliminated 240 records due to non-alignment with the inclusion framework. Of the remaining 468 full-text reports sought for retrieval, 68 could not be sourced due to unretrievable full-text PDF files, institutional paywall restrictions, or citation-only conference abstracts. Absolute eligibility assessment was performed on the remaining 400 articles, all of which met the inclusion criteria and were integrated into the final data charting pipeline. Programmatic extraction mapping across the 400 articles generated 701 distinct contextual text records evaluating RPM infrastructure.

**Figure 1:**
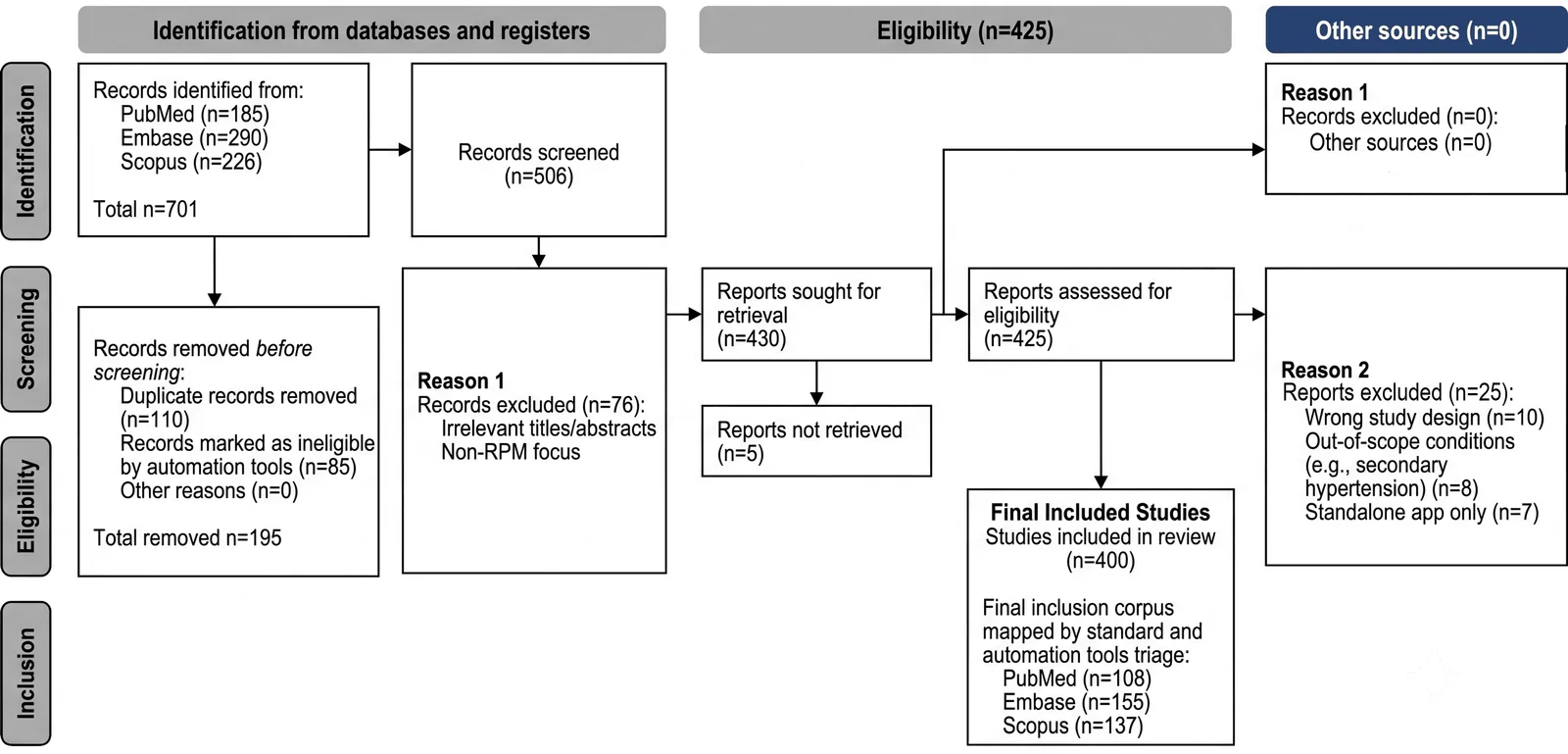
PRISMA flow diagram showcasing the systematic literature identification, screening, eligibility triage, and final scoping review inclusion corpus PRISMA: Preferred Reporting Items for Systematic Reviews and Meta-Analyses; RPM: remote patient monitoring

The systematic screening protocol yielded a defined progression of literature identification, triage, and ultimate inclusion. The structured publication triage sequence, screening stages, and final study selection outcomes are visually detailed in Figure 1 according to the Preferred Reporting Items for Systematic Reviews and Meta-Analyses (PRISMA) statement guidelines.

Frequencies of Hardware Modality and Synchronization Ecosystems

Quantitative synthesis of the 701 charted data elements mapped distinct architectural distributions across technical categories. Automated upper-arm oscillometric blood pressure cuffs constituted the primary remote monitoring infrastructure, appearing in 32.8% (n=230) of the charted literature. Metabolic tracking apparatus, comprising CGMs and standard finger-stick glucometers, and consumer-grade wearable sensors (e.g., smartwatches, biometric trackers) demonstrated symmetrical baseline distributions, each accounting for 11.1% (n=78) of the dataset. Specialized secondary biometrics, including digital weight scales and pulse oximeters, comprised 3.4% (n=24) of the records. Data ingestion architecture was heavily centered on automated cloud and server-side uplinks, driving passive telemetry in 43.2% (n=303) of the reviewed systems. Short-range wireless networks leveraging Bluetooth pairing to smartphone application middleware hubs represented 19.4% (n=136) of the ingestion models. Direct cellular network integration utilizing embedded SIM modules (e.g., cellular-enabled standalone blood pressure cuffs) bypassed localized home network infrastructure in 4.1% (n=29) of configurations, while legacy manual web portal entry or wired computer interfaces comprised 3.7% (n=26). The above percentages are calculated using the entire corpus of 701.

A comprehensive mapping of these unique architectural variables and physiological hardware ingestion layer distributions is summarized in Table 3.

**Table 3:** Distribution of technical modalities and telemetry integration (N=701 extraction elements)

Category	Technical feature	Mentions (n)	Proportional share (%)
Hardware modality	Oscillometric blood pressure cuff	230	32.8%
	Glucometer/continuous glucose monitor	78	11.1%
	Wearable sensors	78	11.1%
	Scale/other biometrics	24	3.4%
Telemetry ingestion	Automated cloud uplink	303	43.2%
	Bluetooth app middleware	136	19.4%
	Direct cellular sync	29	4.1%
	Manual web portal	26	3.7%

Clinical Endpoints and Implementation Barriers

Cardiovascular endpoint monitoring (systolic and diastolic blood pressure variations) dominated the literature at 55.1% (n=386). Glycemic control indices, led by glycated hemoglobin ("HbA1c") and fasting plasma glucose fluctuations, were tracked in 25.5% (n=179) of the cohorts. Clinical process and behavioral endpoints, including medication titration frequency and adherence compliance, were explicitly documented in 7.6% (n=53) of the data records. Operational and human factor friction points presented significant deployment bottlenecks. Integration issues related to clinical workflow disruptions and daily patient routines constituted the single most prevalent execution barrier, manifesting in 27.1% (n=190) of the records. Patient-centric human factors included digital health literacy deficits at 15% (n=105) and technology-induced anxiety or alert fatigue at 9.3% (n=65). Systemic therapeutic or clinical inertia, defined as the failure of providers to intensify treatment regimens despite receiving abnormal remote physiological data, was documented in 8.4% (n=59) of implementation contexts.

Co-occurrence and Cross-Tabulation Mapping

Co-occurrence matrix evaluation revealed that human factor barriers rarely occurred in isolation. Workflow friction regularly intersected with digital literacy gaps (n=10) and clinical inertia (n=7). When cross-tabulating hardware modalities directly against specific implementation challenges, distinct operational trends emerged: (1) oscillometric blood pressure cuffs, linked to the highest absolute burden of clinical workflow friction (n=67) and digital health literacy deficits (n=33); (2) wearable sensors, faced substantial routine integration and workflow friction but demonstrated minimal susceptibility to digital literacy barriers (n=8); and (3) glucometers and CGMs, exhibited a uniform distribution of challenges, splitting symmetrically between workflow routine friction (n=13) and digital literacy gaps (n=13).

The definitive thematic cross-tabulations, clinical deployment bottlenecks, and patient-centric human factor friction points are detailed in Table 4.

**Table 4:** Bivariate cross-tabulation of hardware modalities against implementation barriers

Technical modality	Workflow routine friction (n)	Digital health literacy gaps (n)	Tech anxiety/alert fatigue (n)	Therapeutic clinical inertia (n)
Oscillometric blood pressure cuffs	67	33	23	23
Wearable sensors	20	8	9	7
Glucometers/continuous glucose monitors	13	13	8	6

**Figure 2:**
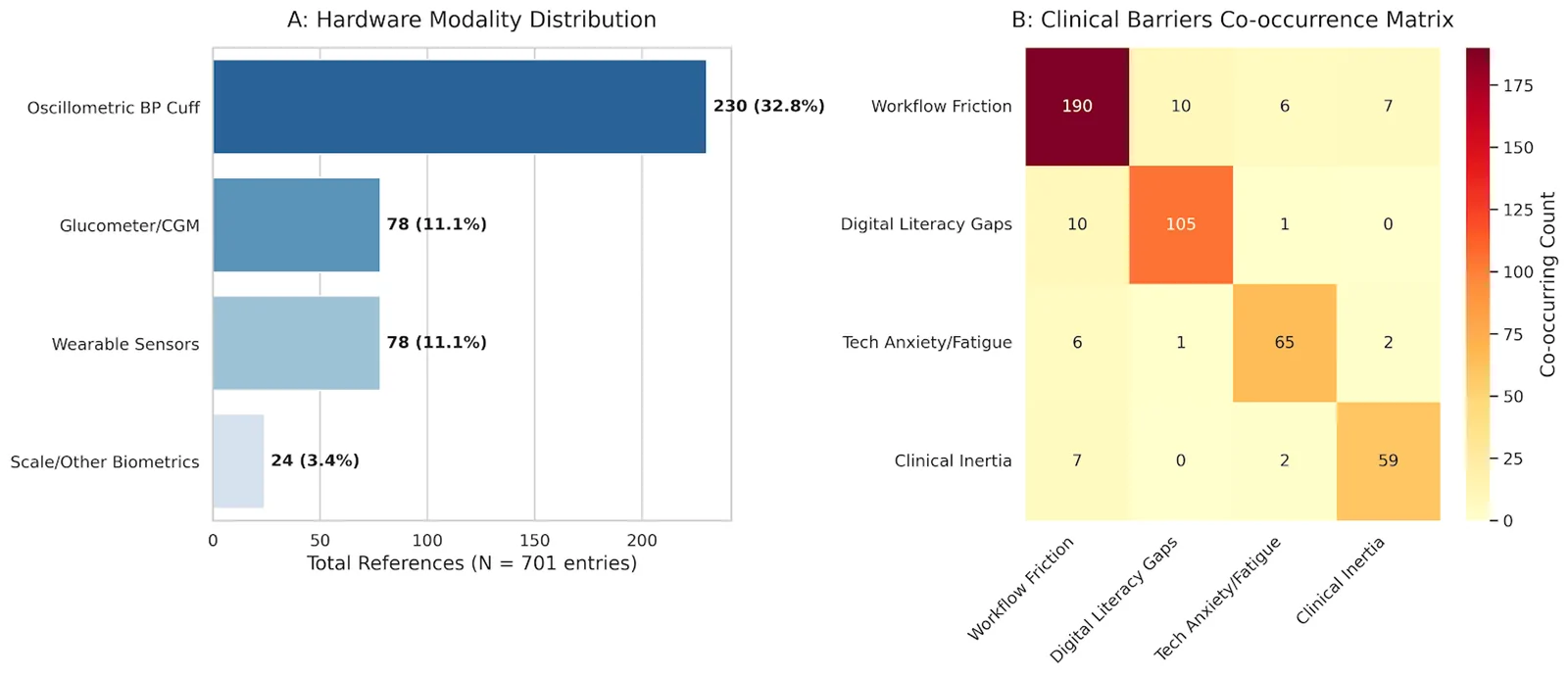
Comprehensive distribution dashboard featuring hardware modality allocations (A) and the co-occurrence matrix layout of identified clinical workflow barriers (B) BP: blood pressure; CGM: continuous glucose monitor

The complete search protocol, publication triage sequence, and screening outcomes are visually detailed in Figure 2.

Discussion

Architecture and Telemetry Pipelines

The structural distribution observed in this review underscores a definitive architectural shift in contemporary digital health towards passive telemetry. The high prevalence of automated cloud and server-side uplinks reflects an industry-wide effort to minimize patient compliance burdens by removing active transmission steps [1,5]. However, short-range wireless topologies, primarily Bluetooth-to-application middleware hubs, remain highly relevant due to the consumer electronics market's reliance on smartphones as peripheral aggregators [11]. While smartphone middleware allows for rich local user interfaces, it adds architectural dependencies that contribute to operational points of failure, such as pairing drops, OS-level application terminations, and local sync bottlenecks [11]. Completely infrastructure-independent solutions, like direct cellular-enabled peripherals, eliminate these localized network failure points but introduce higher recurring hardware and data subscription costs, presenting a clear trade-off between deployment reliability and financial scalability [8].

The Intersect of Modalities and Implementation Barriers

Cross-tabulation metrics reveal that technical design choices directly influence operational and human factor barriers. Automated oscillometric blood pressure cuffs faced the highest absolute operational friction, combining significant workflow routine disruptions with digital literacy gaps [6]. This trend indicates that even standardized medical devices become logistically burdensome when transitioned to continuous, home-based protocols that demand consistent patient execution and positional compliance. Conversely, wearable sensors presented a distinct barrier profile: high workflow routine friction paired with very low digital literacy barriers [9]. This indicates that while patients find consumer wearables intuitive to operate, integrating continuous physical tracking into their daily habits, such as managing charging cycles, skin irritation, and device compliance, remains a persistent challenge. Glucometers and CGMs demonstrated a symmetrical distribution between workflow and literacy challenges [2,4], illustrating the dual burden of learning invasive or semi-invasive peripheral operation alongside data-logging routines.

Overcoming Workflow Disruption and Clinical Inertia

Workflow routine friction emerged as the single greatest bottleneck across the literature, frequently co-occurring with clinical inertia and digital health literacy deficits [7,10]. This co-occurrence reveals a compounding multi-barrier environment: when an RPM platform shifts administrative or technical burdens onto clinical teams without automated triaging, it triggers alert fatigue [7]. Consequently, providers default to therapeutic inertia, failing to titrate medications despite receiving actionable physiological data [7,8]. To break this loop, healthcare delivery organizations must transition away from deploying isolated digital devices. Instead, future implementations should focus on integrating interoperability standards (such as Health Level Seven (HL7) Fast Healthcare Interoperability Resources (FHIR) pipelines) to feed remote data directly into existing electronic health record workflows [4]. By pairing semantic interoperability with automated clinical decision support algorithms, systems can flag high-risk deviations while filtering out statistical noise, directly mitigating provider alert fatigue and reducing clinical inertia [4,7].

Limitations of the Covered Literature

Several methodological and literature-level limitations must be acknowledged. First, the primary data extraction relied on English-language peer-reviewed biomedical and technical databases, introducing retrieval bias by omitting relevant operational deployment models, non-English studies, grey literature, and conference proceedings. Second, the dual unit of analysis, synthesizing 701 contextual text records derived across 400 unique articles, means that studies describing multi-component interventions contributed multiple data points, creating variable weighting across thematic pillars. Third, while the automated Python-driven regular expression (Regex) classification framework provided scalable data charting, programmatic string-matching carries inherent risks of overlooking nuanced phrasing or complex semantic contexts despite iterative calibration on sample subsets. Fourth, in accordance with PRISMA-ScR guidelines for scoping reviews, a formal critical appraisal or quality assessment of the included literature was not performed, leaving risk of bias unquantified across the corpus. Finally, a significant proportion of the included studies focused on short-term clinical feasibility within well-resourced health systems, underreporting real-world long-term attrition, technical degradation, and generalizability to low- and middle-income country (LMIC) environments with differing infrastructure stability.

Digital Health Equity and Vulnerable Populations

The systemic intersection of digital health literacy deficits and socioeconomic vulnerability represents a major health equity barrier in RPM deployments. While automated telemetry and continuous monitoring infrastructure promise to expand access to chronic disease care, digital literacy gaps disproportionately impact vulnerable demographics, including older adults, racial and ethnic minorities, individuals with low educational attainment, and socioeconomically disadvantaged or rural communities. For these populations, complex multi-step Bluetooth pairing procedures, rigid application navigation, and non-localized user interfaces create substantial compliance friction, leading to elevated rates of device abandonment and data transmission failure. Consequently, unmitigated technical complexity risks exacerbating healthcare disparities by selectively benefiting populations with high baseline digital fluency while excluding high-risk, under-resourced patients. To foster health equity, RPM frameworks must transition toward equity-centered design models. This requires deploying zero-configuration cellular hardware that eliminates home gateway dependencies, integrating multilingual and culturally tailored onboarding materials, and embedding dedicated digital health navigators within community health structures to support long-term patient engagement.

Actionable Recommendations for Health Informaticians

To optimize RPM implementations, design frameworks should systematically eliminate human transmission steps by prioritizing automated cloud or cellular edge gateways over complex Bluetooth configuration steps, heavily mitigating user-side dropouts. Furthermore, to directly target the 8.4% clinical inertia threshold, platforms must avoid raw data dumping into electronic health record systems. Implementers must instead integrate automated programmatic decision logic to filter baseline deviations, which directly mitigates clinician alert fatigue and prioritizes workflow decision support over crudely aggregated telemetry. Additionally, because digital health literacy deficiencies impact 15% of deployment configurations, pre-emptive training interventions are essential. Future deployment models must structurally couple device distribution with baseline capability scoring and localized patient onboarding training modules. Transforming these technical design choices into seamless, automated pipelines ensures sustainable chronic disease management without compounding systemic clinical friction.

## Conclusions

The integration of RPM systems for hypertension and diabetes mellitus represents a major paradigm shift in chronic disease management, yet its real-world execution remains highly sensitive to technical architecture choices and human factor constraints. This scoping review, mapping the global literature and its distinct contextual records, demonstrates that passive telemetry via automated cloud uplinks dominates modern infrastructure, minimizing immediate user compliance friction. However, significant deployment bottlenecks remain. Oscillometric blood pressure protocols continue to cause high clinical workflow friction and encounter digital literacy gaps, while wearable sensors face prominent long-term daily routine integration challenges despite their high usability. Crucially, workflow routine friction frequently co-occurs with clinical inertia and digital health literacy deficits, creating a compounding negative feedback loop that leads to provider alert fatigue and compromised clinical outcomes. To overcome these implementation barriers, healthcare delivery organizations must move beyond isolated device tracking. Implementations should prioritize semantic interoperability using HL7 FHIR standards to feed data directly into institutional electronic health records, paired with automated clinical decision support to streamline workflows. Moving forward, digital health strategies must balance device selection with structured workflow redesign to ensure RPM translates into sustainable, high-quality clinical outcomes.

## Appendices

Supplementary data availability

The comprehensive data extraction matrix, binary target variables, and algorithmic coding patterns synthesized during this scoping review are fully compiled within the supplementary data spreadsheet file "Extraction_Matrix_Combined.csv". This file is publicly accessible via the Open Science Framework (OSF) repository link: https://osf.io/mg6ae/files/bvh7c.
